# Echovirus 30 and coxsackievirus A9 infection among young neonates with sepsis in Iran

**Published:** 2018-08

**Authors:** Abdolnabi Shabani, Manoochehr Makvandi, Alireza Samarbafzadeh, Ali Teimoori, Mojtaba Rasti, Chiman Karami, Nasteran Rastegarvand, Roya Nikfar, Ahmad Shamsizadeh, Azam Salehi, Kambiz Ahmadi Angali

**Affiliations:** 1 Infectious and Tropical Diseases Research Center, Health Research Institute, Ahvaz Jundishapur University of Medical Sciences, Ahvaz, Iran; 2 Department of Infectious Diseases, Abozar Hospital, Ahvaz Jundishapur University of Medical Sciences, Ahvaz, Iran; 3 Parasitology Department, Ahvaz Jundishapur University of Medical Sciences, Ahvaz, Iran; 4 Biostatistic Department, School of Health, Ahvaz Jundishapur University of Medical Sciences, Ahvaz, Iran

**Keywords:** Sepsis, Enteroviruses, Echovirus, Coxsackievirus

## Abstract

**Background and Objectives:**

Human enteroviruses (EV) are the most common causes of neonatal sepsis-like disease. The frequencies of EV including coxsackievirus A, coxsackievirus B and Echovirus serotypes have been studied in young infants (younger than three months) with sepsis. So far, the role of enteroviruses among neonates with sepsis was not determined in Ahvaz, Iran. Therefore, this study was aimed to evaluate the frequency of EV among hospitalized young infants with clinical signs and symptoms of sepsis in Ahvaz.

**Materials and Methods:**

Blood specimens from 128 neonates (younger than 90 days), including 56 (43.75%) girls and 72 (56.25%) boys, were collected from hospitalized neonates with clinical signs and symptoms of sepsis-like symptoms. All blood samples were negative for bacterial culture. RNA was extracted from all sera and tested for detection of 5′UTR (Untranslated Region) of the EV by RT-PCR. To determine specific strains of EV, positive 5′UTR samples were further tested for detection of the VP1 region of EV by RT-PCR.

**Results:**

Overall, 50/128 (39.06%) specimens, including 24 (48%) girls and 26 (52%) boys, were positive for EV. 21/50 (42%) specimens were positive for the VP1 region. Randomly, 8 positive VP1 were selected and sequenced. Analysis of sequencing data showed 7/21 (33.33%) samples were positive for Echovirus 30 and 1/21 (4.76%) samples were positive for CVA9.

**Conclusion:**

The results of this survey indicate high prevalence of 39.06% of EV among young neonates with sepsis. A high prevalence of 33.3% Echoviruses 30 and a low rate of 4.76% coxsackievirus A9 infection has been observed in neonatal patients with viral sepsis. This outbreak is probably one of the first Enterovirus outbreaks to be reported in Ahvaz, Iran. The results of this survey will help to minimize unneeded use of antimicrobial drugs and reduce unnecessary hospitalization.

## INTRODUCTION

Sepsis symptoms are non-specific and include abdominal distention, apnea, fever, irritability and fatigue, seizures, rash, and feeding problems. The infectious agents of neonatal sepsis are either viral or bacterial (1–7). Among the viruses the Human Enteroviruses are the important agents to cause sepsis in young neonates worldwide. Human Enteroviruses (EVs) genus are single positive RNA and belong to picornaviridae (1) Based on their antigenic and pathogenic properties in humans and laboratory animals, EVs are classified into polioviruses (PV, three serotypes), Coxsackie A viruses (CAV, twenty three serotypes), Coxsackie B viruses (CBV, six serotypes) and echoviruses (E, twenty eight serotypes) and Enteroviruses 68–71 (2). However, this biological division has been replaced by a molecular classification based on VP1 sequencing (3). The sequence analysis of human enteroviruses were furthermore grouped into four species (EV-A to D) among them six different species A (CAV2, CAV4, CAV6, CAV10, CAV16 and EV71) and four serotypes species B (E6, E9, E30 and CAV9) have been detected in young children with sepsis- like illness in Edinburgh and United State of America (4–6). Within EV species B (all echoviruses, CBV1-6 and CAV9) have been detected in neonatal with sepsis- like illness (1–4). Human enteroviruses have been classified into more than 100 serotypes, among them all echoviruses, coxsackie-virus A9 (CVA9), enterovirus 69 and enterovirus 71 are well known agents for meningitis (8, 9). Human enterovirus is associated with a wide range of diseases from febrile to multi-systemic illnesses like aseptic meningitis, paralysis, myocarditis and neonatal sepsis (10). EVs can be transmitted both horizontally (fecal-oral/oral-oral) and vertically (prenatal infection). Human Enteroviruses (EV) are a common cause of neonatal sepsis especially at the junction of summer and fall. Infection occurs in young infants especially in summer and autumn (11). The rate of viral pathogens in febrile infants (1 to 90-day-old) is 3 times of bacteria (12). The epidemiology of viral sepsis among the young neonates have been reported in Kuwait (3), Scotland (4), England (5), USA (6), Palestine (8) and the Netherlands (11).

Laboratory detection of EV generally is carried out by reverse transcription PCR (RT-PCR), or Real time-PCR which are faster and more sensitive than viral cell culture. The 5’non-coding region (NCR) is the most conserved region among EV, and target in many diagnostic screening procedures (13). However, sequences from this region provide little or no information on the serotype of the infecting virus, and sequencing of a structural gene region such as VP1 is required to identify EVs and Human Parecho virus (HPeV) (4, 13). This method is used for detecting enterovirus in sepsis and meningitis patients (13, 14). VP1 is one of the capsid’s proteins which contains a serotype-specific antigenic neutralization site. Moreover VP1 sequence comprises hyper variable region, high rate of mutation and, recombination, thus the analysis of this region is used in determination and phylogenetic classification of enterovirus serotypes (15, 16). Since the knowledge regarding the role of enteroviruses in young neonates with sepsis is limited in our region, therefore, this study was aimed to determine the etiologic agents of viral sepsis and their molecular characterization in young neonates hospitalized in Ahvaz city, Ahvaz city is capital of Khozestan province and located in southwest region of Iran.

## MATERIALS AND METHODS

Initially, the sera were collected from 140 young neonates who were hospitalized to Abozar Hospital, Ahvaz city, Iran, During September 2013- October 2014. The patients with age > 3 months, the patient sample positive for bacterial infection were excluded. The blood culture was done for all samples. Twelves samples were positive for bacterial pathogens and excluded from the study. The inclusion criteria were patients aged <90 days, fever >38°C with irritability and tachycardia. In Total, 128 neonates, including 56 (43.75%) females and 72 (56.25%) males were recruited for this study. The demography of patients including sex, age, clinical symptoms (fever, Tachycardia), laboratory parameter such as WBC count, platelet, CRP (C - reactive protein) was recorded for each individual.

### RNA extraction and RT-PCR.

RNA was extracted from 128 sera samples using High Pure RNA Isolation kit (Roche/Germany) according to manufacturer’s protocol. The cDNA was prepared based on the supplier’s instruction (Thermo scientific/USA). Detection of enterovirus genome was carried out by a semi-nested PCR. The Sabin vaccine was used as positive control. The PCR reaction mixture was comprised of 2.5 μl PCR buffer, 0.5 μl dNTP, 0.75 μl of each EV-F1 and EV-R primers (Table 1), 0.75 μl MgCl_2_, 0.12 μl Taq, 2.5 μl template and D/W up to 25 μl. The reaction mixture was subjected to the thermocycler (TC-512, Techne, UK) for 35 cycles of amplification (94°C for 45 s, 54°C for 45 s, 72°C for 45 s and 72°C for 10 min). Then, 1 μl of the product was used as the template for the second round which consisted of 2.5 μl PCR buffer, 0.5 μl dNTP, 0.75 μl of each EV-F2 and EV-R primers, 0.75 μl MgCl_2_, 1 μl template, 0.12 Taq and 18.62 μl D/W was subjected for 35 cycles of amplification (94°C for 30 s, 58°C for 30 s, 72°C for 30 s and 72°C for 5 min). The final product of 155bp electrophoresed on 2% gel indicated the positive test (17).

**Table 1.** Universal primers of the semi nested PCR

**Primer**	**Genome region**	**Sequence**	**Product size**	**Reference**
First Step	5′ UTR	EV-1 (F1):CAAGCACTTCTGTTTCCCCGG	440 bp	(17)
		EV-R: ATTGTCACCATAAGCAGCCA		
Second Step	5′ UTR	EV2 (F2):TCCTCCGGCCCCTGAATGCG	155 bp	(17)
		EV-R: ATTGTCACCATAAGCAGCCA		
	VP1	VP1 (F):GCRTGCAATGAYTTCTCWGT	1000 bp	(18)
		VP1 (R):GCICCIGAYTGITGICCRAA		

Detection of the VP1 region of Enterovirus was carried out for those samples positive for 5UTR. The PCR reaction mixture was comprised 2.5 μl 10X PCR buffer, 0.5 μl dNTP, 0.5 μl of each VP1-F and VP1-R primers (Table 1), 0.12 μl Taq, 2.5 μl template 0.5 μl dNTP and water up to 25 μl was subjected to thermocycler for 35 cycles of amplification (94°C for 45 s, 54°C for 45 s, 72°C for 45 s and 72°C for 10 min). The final product of 1000bp electrophoresed on the 2% gel indicated the positive test.

### Sequencing.

To determine EV serotypes, randomly, 8 positive samples of the VP1 region were sequenced. The sequences were blasted using available databases. A phylogenic tree was constructed with the Neighbor joining method using the partial nucleotide sequences of the VP1 region of EV positive samples. Reference sequences were retrieved from the GenBank using their accession numbers.

### Ethic status.

This project with the registration number ajums.REC.1392.338 was approved in the ethic committee of Ahvaz Jundishapur University of Medical Sciences Ahvaz, Iran. Consent was obtained from each young neonate parent.

### Statistical analysis.

Statistical analysis was carried out for the variables WBCs, neutrophils, platelet and C-Reactive Protein. The variables were recorded as means, standard deviation. To analyze the association of EV in genders, age group and seasons were performed by chi-square or Fisher exact test. Data were analyzed by SPSS Statistics ver. 22. *P* Values <0.05 were considered to be statistically significant.

## RESULTS

**Fig. 1.**
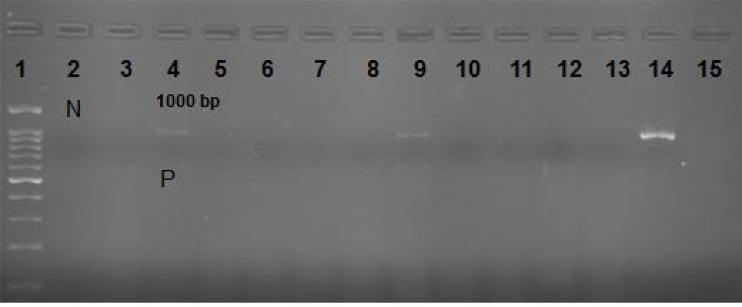
VP1 PCR. N: Line 1: 100 bp DNA ladder, Line 2 N: Negative control; Line 4, P: Positive control; # 3, 6 to 14: Unknown samples

**Fig. 2.**
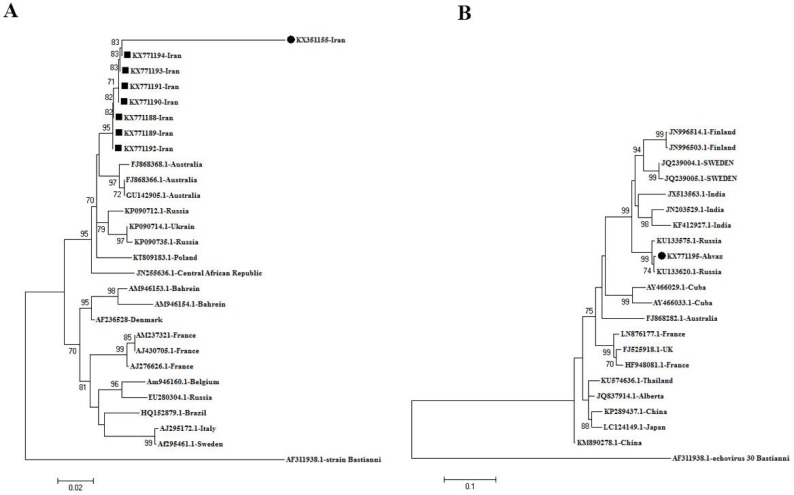
A: Neighbor-joining phylogenetic tree was drawn with MEGA software version 6 of the nucleotide sequences in the VP1 regions of the strains of Echo 30 isolated from patients with sepsis in Ahwaz-Iran with accession number (KX771182-94) retrieved from Gene Bank and compared with other Echo 30 isolated from other regions of the world. Iran (Ahvaz) isolates are labeled by black solid squares and, whereas the Echo 30 (KX351155) Iran (Ahvaz) prototype strain is labeled by the black solid circle. They were compared with other Echo 30 strains isolates from other regions of the world. Numbers in branches are reproducible after 1,000 bootstraps. Scale bar=2%. B: Neighbor-joining phylogenetic tree of the nucleotide sequences in the VP1 regions of the strain of coxsackievirus A9 isolated from a patient with sepsis in Ahvaz, Iran with accession number KX771195 labeled by the black solid circle. It was compared with other coxsackievirus A9 isolates from other regions of the world, Numbers in branches are reproducible after 1,000 bootstraps. Scale bar=0.1

**Graph 1.**
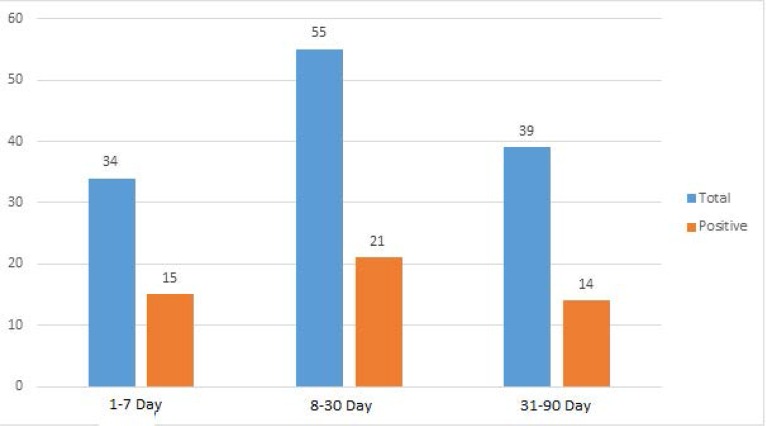
Distributions of Enterovirus among different age groups.

**Graph 2.**
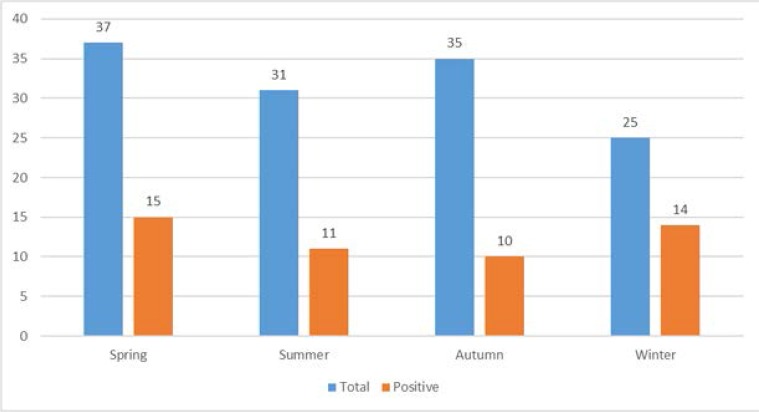
Frequency of Enterovirus in different seasons. The GenBank accession numbers for VP1 sequences were as follows: KX771188, KX771189, KX771190, KX771191, KX771192, KX771193 and KX771194 and KX771195.

All 128 samples were negative for the bacterial pathogen. The Enterovirus genome was detected in 50 (39.06%) specimens including 24 (48%) females and 26 (52%) males by PCR. Of 50 specimens, 21 specimens showed positive for VP1 region. The sequencing analysis of 8 samples for VP1 revealed that 7 samples were Echovirus 30 and one sample was coxsackievirus A9 (Fig. 1). Table 2 shows distribution of enterovirus relative to gender, age, different seasons, clinical symptoms and laboratory parameters. Graph 1 and 2 show the distribution of Enterovirus among different age groups and seasons, The neighbor-joining phylogenetic tree was drawn with MEGA software version 6. The nucleotide sequences in the VP1 regions of the strains of Echo 30 virus isolated from patients with sepsis in Ahvaz-Iran with accession number (KX771182-94) labeled by black solid squares (Fig 2. A). Neighbor-joining phylogenetic tree of the nucleotide sequences in the VP1 regions of the strain of coxsackievirus A9 isolated from a patient with sepsis in Ahvaz, Iran with accession number KX771195 labeled by the black solid circle (Fig 2. B).

**Table 2.** Profile of neonates sepsis patients associated with EV.

**Characteristic**	**EV positive**	**%**	***P* value**
No. of patients (n=128)	50	(39.06%)	
**Sex**			
Female	24/56	(18.75%)	0.438
Male	26/72 (20.31%)		
**Age (days)**			
1–7	15/34	(44.11%)	
8–30	21/55	(38.18%)	0.48
31–90	14/39 (35.89%)		
**Season**			
Winter	14/25	(56%)	0.035
Spring	15/37	(40.54%)	
Summer	11/31	(35.48%)	
Autumn	10/35	(28.57%)	
**Clinical symptoms**			
Fever 38–39.5	128	(100%)	
Tachycardia	39/128	(30%)	
**Laboratory parameter**			
WBC (/mm^3^)	NA	9,790 - 28000	
Absolute neutrophil count (/mm^3^)	NA	4,362 - 6,955	
Platelet (×10^3^/mm^3^)	NA	313–450	
CRP C-reactive Protein (mg/L)	NA	3.5±1.7	

NA, Not Applicable

Table 2. Frequency of EVs among males and females was not significant (*p*=0.438). Distribution of EVs among different age groups was not significant (*p*=0.48), Frequency of EVs in winter was higher than other seasons (*p*= 0.035)

## DISCUSSION

Sepsis in neonates and young infants is an important clinical entity that result in large numbers of hospital admissions annually. It is estimated that about 0.6%–3% of infants are readmitted to the hospital for suspected sepsis in the first month of life, and the majority of the patients are infected with enterovirus (19).

This outbreak is probably one of the first large Enteroviruses outbreaks to be reported in Ahvaz city, Iran. The results of this survey will help to minimize unneeded use of antimicrobial drugs and reduce unnecessary hospitalization. Syndromic surveillance that used emergency department data proved to be a useful and proper way to monitor emergency department hospital admissions associated with the Enteroviruses outbreak.

Our study described the frequency of EV infections in the sera of young neonates suspected sepsis, in Ahvaz, Iran, from September 2013 to October 2014. In the present study the Enterovirus genome was detected in 50/128 (39.06%) specimens including 24 (18.75%) females and 26 (20.31%) males (*p*=438). The EV detection rate in young neonates varied in different regions of the world.

Harvala et al. in Edinburg, investigated this phenomenon over eight-years and reported 58% of young neonates with sepsis were positive for EV (4). Maguire et al. have detected enteroviruses in 29% of young neonates with sepsis in England and Wales (5). Khetsuriani et al. have detected EV in 44% of young neonates with sepsis in USA which is in agreement with our finding (20).

Verboon-Maciolek et al. diagnosed EVs in 57% of neonates with sepsis syndrome in Netherlands which shows higher percentages than our finding (11). Jordán from Spain has reported 10/72 (13.88%) of febrile neonates were positive for EVs which indicate lower percentages than our finding (21). In our study, 7/8 (87.5%) of the neonates with sepsis were found positive for Echo virus 30, while 1/8 (12.5%) cases were positive for coxsackievirus A9. Both Echo virus 30 and coxsackievirus A9 belong to Enteroviruses B species and were associated with sepsis patients. The role of Enteroviruses B species in young neonates with sepsis have been reported by other investigators (4–6). The frequency of Echo virus 30 was found dominant among the young neonates in our region. Harvala et al. have described the E9, CAV9, CBV5, E6, E11, E30, CAV2,CAV4, CAV6, CAV10, CAV16 and EV71 as being associated with sepsis- like illness which among them E6, E9, E30 and CAV9 are the most abundant serotypes isolated from clinical specimens in the UK and USA (4, 19, 20). Ji-Hyun Seo et al. have described detection of enterovirus among young children with a sepsis-like illness to be 7.5% in Jinju, Korea (22). It should be considered that the reason for discrepancy in prevalence between different other studies and this may be due to the sensitivity of the method used or types of samples.

In our study detection of EVs was observed among young neonates with sepsis in all seasons, autumn (28.57%), winter (56%), spring (40.54%) and summer (35.48%). The peak EV detection was observed in the winter season while the lowest detection was in autumn (28.57%) (*p*=0.035). Our finding show that HEV infection occurs throughout the year and not restricted to spring and summer, whereas, Harvala et al. 2011 (Edinburgh, United Kingdom) and Ju-Young Chung et al. 2015 (Jinju, south Korea) have described the peak EV detections to be in spring (22, 23). Han et al. 2013 (Seoul, Korea) and Walters et al. (Chicago, United States) have described the peak season for EV detection was in summer (24, 25).

In Iran, the frequency of enterovirus in neonates was reported in Mashhad city to be 37% (26) and in Tehran to be 32% (27). Rasti et al have described the detection of EVs in children under one-year of age with aseptic meningitis as 55.8% in Ahvaz (28).

In the present study, 78/128 (60.93%) of the patients with sepsis- like syndrome were shown to be negative for EVs. The role of parechoviruses have been recognized as important agents of sepsis in young neonates (29, 30, 31). Among them the role of parechovirus 3 in association with sepsis- like syndrome in the young neonates is more dominant (29, 30). In the present study, the patients' serum was used for detection of EV. Serum is as good as or better than CSF as a diagnostic tool for systemic enterovirus infection, although more patients should be analyzed to confirm this observation. A rapid diagnosis of enterovirus infection in young infants is important to prevent and reduce broad-spectrum antibiotics use, and possibly to indicate that anti-viral treatment should be initiated, in severe cases. Rapid diagnosis of enterovirus disease has been demonstrated to significantly reduce hospital stay and costs (19).

## CONCLUSION

The results of this survey indicate the frequency of EV among the patients with sepsis was 39.06%. Among the positive EV cases, high frequency of 14% Echoviruses 30 and low prevalence of 2% coxsackievirus A9 were observed in young neonates with viral sepsis. 78 (60.93%) of the patients were shown negative for EV. The role of parechovirus needs to be investigated. Syndromic surveillance is a useful proxy indicator that should be considered for future detection and surveillance of seasonal outbreaks of Enterovirus infections. This outbreak is probably one of the first Enterovirus outbreaks to be reported in Ahvaz, Iran. The results of this survey will help to minimize unneeded use of antimicrobial drugs and reduce unnecessary hospitalization.
